# Correlation Between Postoperative Early Recurrence of Hepatocellular Carcinoma and Mesenchymal Circulating Tumor Cells in Peripheral Blood

**DOI:** 10.1007/s11605-017-3619-3

**Published:** 2017-11-20

**Authors:** Zhong Wang, Lei Luo, Yuan Cheng, Guolin He, Bangjian Peng, Yi Gao, Ze-sheng Jiang, MingXin Pan

**Affiliations:** Second Department of Hepatobiliary Surgery, Zhujiang Hospital, Southern Medical University, Guangzhou, 510282 China

**Keywords:** Hepatocellular carcinoma (HCC), Epithelial-mesenchymal transition (EMT), Circulating tumor cells (CTCs), Early recurrence(ER)

## Abstract

**Background:**

Circulating tumor cells (CTCs) have been actively studied for their functions in hepatocellular carcinoma (HCC) recurrence. However, the relationship between circulating tumor cells subtypes and hepatocellular carcinoma recurrence is still unclear.

**Methods:**

CTCs were collected from the peripheral blood of 62 postoperative HCC patients. The CTCs were isolated with a filtration-based method. Multiplex fluorescence in situ hybridization was used to characterize the CTCs based on mRNA expression levels of epithelial and mesenchymal markers.

**Results:**

Of the 62 HCC patients, 26 were diagnosed with early recurrence (ER) and 36 did not experience recurrence. Comparison between the recurrence group and the non-recurrence group showed the total number of CTCs, mesenchymal CTCs, and mixed CTCs in the recurrence group was significantly higher than in the non-recurrence group. Receiver operator characteristic (ROC) curve analysis was performed to define the positive cutoff values as follows: total number of CTCs ≥ 4, mesenchymal CTCs ≥ 1, and mixed CTCs ≥ 3. Analysis showed that portal vein tumor thrombus (hazard ratio [HR] = 2.905, *P* = 0.023) and mesenchymal CTC positivity (HR = 3.453, *P* = 0.007) were independent risk factors for ER. The correlation between the presence of mesenchymal CTCs and time to recurrence was further examined, and the results showed significantly shortened postoperative disease-free survival in patients positive for mesenchymal CTCs (*P* < 0.001).

**Conclusions:**

HCC patients with positive peripheral mesenchymal CTCs have a more serious risk of ER, which could be a potential biomarker in HCC prognosis monitoring.

## Introduction

Hepatocellular carcinoma (HCC) is one of the most common malignancies in the world and has high morbidity and mortality. In recent years, the incidence of HCC has continued to rise, especially in developing countries. Furthermore, the prognosis of HCC patients is still not optimistic, with a 5-year survival rate of less than 20%. Nowadays, surgical resection is the preferred treatment for most HCC patients. However, postoperative recurrence is the main factor that affects HCC prognosis.^1^,^2^ Currently, monitoring HCC recurrence mainly relies on ultrasonography, computed tomography (CT) examination, and serum alpha-fetoprotein (AFP) levels.^3^ However, these conventional monitoring methods cannot detect early recurrence in most cases. Therefore, it is crucial to identify sensitive biomarkers for the early diagnosis of HCC recurrence, which will facilitate the early detection of HCC recurrence and timely interventions to improve survival.

In 1896, Ashworth, an Australian researcher, found a type of cells that was very similar to tumor cells in the blood of a patient with a metastatic tumor, and he proposed the concept of circulating tumor cells (CTCs).^4^ Tumor recurrence and metastasis are very complex processes, which are usually considered to require CTC migration from the primary tumor focus into the peripheral blood.^5^ In addition, because peripheral CTC detection is a simple, reproducible, and minimally invasive procedure, CTCs have been actively studied over the last few decades for their functions in tumor diagnosis, recurrence, and metastasis.^6^–^9^ However, studies on the relationship between CTC subtypes and tumor recurrence have rarely been reported. In this study, peripheral CTC subtypes were examined in patients with HCC, and their relationship with HCC recurrence was analyzed. The clinical value of detecting each subtype of CTCs for early prediction of HCC recurrence was also investigated.

## Materials and Methods

### Patient Selection

Sixty-two hepatocellular carcinoma patients (58 males and 4 females 30–79 years old, with a median age of 55) who underwent radical resection at Zhujiang Hospital of Southern Medical University from March 2014 to March 2016 were enrolled in this prospective study. The inclusion criteria were as follows: (1) patients who underwent pathological specimen examination and had a definite pathological diagnosis of liver cancer according to the criteria set by the World Health Organization; (2) patients who underwent radical resection by an experienced physician, with no residual lesions at the margins of the excision site as confirmed via postoperative pathology examination; (3) patients who had not been treated with other anti-tumor therapies before the radical resection; and (4) patients who had no extrahepatic metastasis confirmed by preoperative imaging. Tumor stage was determined according to the Barcelona Clinic Liver Cancer (BCLC) staging classification, and the degree of tumor differentiation was defined according to the Edmondson-Steiner grading system.

### Follow-Up and Recurrence

The patients entered the clinical follow-up period to monitor for recurrence after their peripheral blood samples were collected postoperatively (3–35 days after the surgery, with a median of 18 days). The patients underwent various follow-up examinations and treatments after surgery according to routine clinical schedules. Recurrence was defined as intrahepatic recurrence and extrahepatic metastasis from a comprehensive diagnosis based on imaging results from computed tomography (CT), magnetic resonance imaging (MRI), and digital subtraction angiography (DSA), or positron emission tomography (PET)-CT and serum AFP level and other examinations, with or without pathological diagnosis. Evidence of recurrence was considered as the end point. Time to recurrence (TTR) was defined as the time interval between resection and the diagnosis of recurrence. Recurrence within 6 months after the surgery was defined as ER. Patients with no recurrence were followed up to September 30, 2016.

### Detection and Characterization of CTCs Using the CanPatrol™ System

Five milliliters of peripheral blood sample from each patient was placed in a K2-ethylenediaminetetraacetic acid (EDTA) tube and centrifuged to collect cell pellets. The supernatant was discarded, and 5 mL of phosphate-buffered saline (PBS) was added to the tube to resuspend the cell pellet. The cell suspension was filtered through a filter tube (Surexam Biotech, Guangzhou, China) containing a membrane filter (Millipore, Billerica, MA, USA) with a pore size of 8 μm. The cell suspension was passed through the filter under vacuum, and the circulating tumor cells (CTCs) remained on the filter. As the diameter of blood cells is smaller than the diameter of CTCs, the blood cells passed through the filter.^10^

We designed three sets of nucleic acid probes to detect and characterize the expression levels of epithelial and mesenchymal genes in CTCs through multiplex RNA in situ hybridization (RNA-ISH). The first set of probes contained four epithelial transcripts (CK8, 18, and 19; EpCAM). The second set of probes consisted of two mesenchymal transcripts (Vimentin and Twist). The last set contained only CD45 transcripts to distinguish between leukocytes and CTCs. Detailed procedures for the hybridization followed previously published methods.^11^ Briefly, the cells remaining on the filter were permeabilized and digested with protease followed by a series of hybridization steps using the probes described above. Finally, the nuclei were stained with 4′, 6-diamidino-2-phenylindole (DAPI). The cells were analyzed using fluorescence microscopy. The red and green fluorescent signals observed in the cells represented the expression of the epithelial and mesenchymal genes, respectively. The blue fluorescent dots indicated CD45 expression, which is a marker of leukocytes.^10^,^12^

### Statistical Analysis

The data were statistically analyzed using SPSS 21.0 statistical software. The CTCs and their subtypes were compared between patients with and without recurrence. The measurement data were analyzed using the rank-sum test (the measurement data did not exhibit a normal distribution on the normality test). The results for the predicted rates of recurrence based on different types of CTCs were used to plot receiver operator characteristic (ROC) curves, and the area under the curve (AUC) was calculated. The cutoff point corresponding to the maximum of the Youden index was taken as the best critical point for clinical diagnosis. The Kaplan-Meier test was used to compare the difference in survival rates between groups. The Cox proportional hazards regression model was used to compare the difference in survival rate. All the tests were bilateral, and the significance level was set at *α* = 0.05.

## Results

### Relationship Between CTC Subtype and ER after Radical Resection

To study the relationship between CTC subtypes and ER after radical resection, 62 HCC patients, including 26 patients with ER (aged 30–74 years old with a median age of 52) and 36 patients without recurrence (aged 30–79 years old with a median age of 57.5), were followed up after surgery. Comparison between the recurrence group and the non-recurrence group showed a median of 6 vs 2.5 total CTCs, with 0.5 vs 0 median epithelial CTCs, 3 vs 1 median mixed CTCs, and 1 vs 0 median mesenchymal CTCs. A rank-sum test showed significantly more total CTCs (*P* = 0.011), mesenchymal CTCs (*P* < 0.001), and mixed CTCs (*P* = 0.027) in the peripheral blood of the recurrence group than in the non-recurrence group after HCC radical resection. No difference was observed in the epithelial CTC counts between the two groups (*P* = 0.619) (Table 1).

**Table 1:** The comparison of each CTC subtype count between recurrence and non-recurrence (median and interquartile range)

	Total CTCs	Epithelial CTCs	Mixed CTCs	Mesenchymal CTCs
Non-recurrence (*n* = 36)	2.50 (1.00–8.00)	0.00 (0.00–2.75)	1.00 (0.25–5.00)	0.00 (0.00–0.00)
Recurrence (*n* = 26)	6.00 (3.75–17.25)	0.50 (0.00–2.00)	3.00 (1.75–9.00)	1.00 (0.00–4.00)
*Z*	2.556	0.498	2.213	3.994
*P* value	*0.011*	0.619	*0.027*	*0.000*

*P* < 0.05 was considered statistically significant

### Analysis of Risk Factors for Postoperative HCC Recurrence

**Fig. 1:**
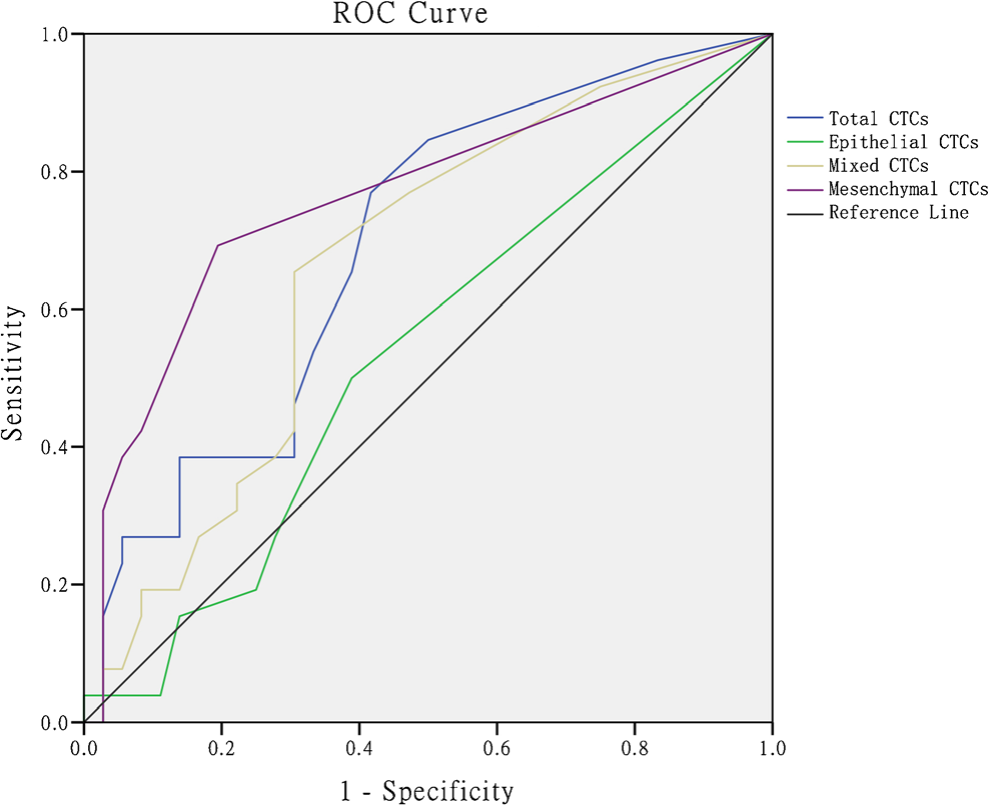
ROC curves of different circulating tumor cell subtypes

The cutoff values for each CTC subtype which was associated with ER were determined via ROC curve analysis, and the cutoff was considered positive for total CTCs ≥ 4, mesenchymal CTCs ≥ 1, and mixed CTCs ≥ 3 (Fig. 1, Table 2).

**Table 2:** Diagnostic values of CTC count at selected cutoff point

CTC subtype	Cutoff point	Sensitivity	Specificity	YI	AUC
Total CTCs	≥ 3	0.846	0.500	0.346	0.691
	≥ 4	0.769	0.583	0.352	
	≥ 5	0.654	0.611	0.265	
Mixed CTCs	≥ 2	0.769	0.528	0.297	0.664
	≥ 3	0.654	0.694	0.348	
	≥ 4	0.423	0.694	0.117	
Mesenchymal CTCs	≥ 1	0.692	0.806	0.498	0.764
	≥ 2	0.423	0.917	0.340	
	≥ 3	0.385	0.944	0.329	

**Fig. 2:**
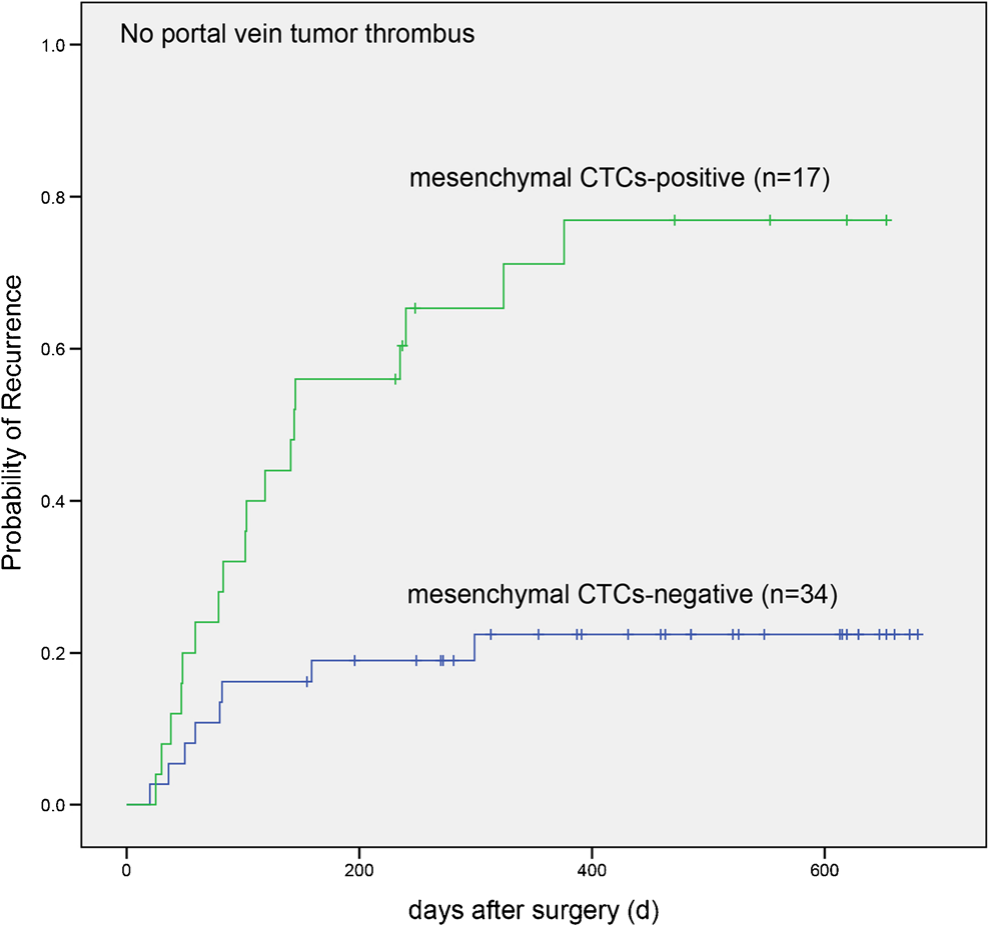
HCC patients without portal vein tumor thrombus: log-rank test for time to recurrence with mesenchymal CTC-positive or negative postoperatively

To systematically analyze the relationship between ER after HCC radical resection and total CTCs, mesenchymal CTCs, mixed CTCs, or other factors, univariate Cox regression analysis was first performed for recurrence and each factor. The results showed a significant correlation between tumor recurrence and mesenchymal CTCs (*P* < 0.001), mixed CTCs (*P* = 0.009), total CTCs (*P* = 0.02), portal vein tumor thrombus (*P* = 0.001), and tumor size (*P* = 0.049). Multivariate Cox regression analysis further showed that mesenchymal CTCs (hazard ratio [HR] = 3.453, *P* = 0.007) and portal vein tumor thrombus (HR = 2.905, *P* = 0.023) were independent risk factors for ER after HCC radical resection (Table 3), suggesting that mesenchymal CTCs have more predictive strength than portal vein tumor thrombus with respect to recurrence outcome. The correlation between mesenchymal CTCs and TTR was examined using the K-M test. Among the patients who were negative for portal vein tumor thrombus, the probability of recurrence was significantly higher in mesenchymal CTC-positive patients than in mesenchymal CTC-negative patients (*P* < 0.001, Fig. 2) over increasing follow-up durations.

**Table 3:** Analyses of risk factors for HCC early recurrence

		Recurrence		Univariate analysis			Multivariate analysis		
		No	Yes	HR (95% CI)	*χ* ^2^	P*	HR (95% CI)	*χ* ^2^	*P*
Age (years)	≤ 50	8	10	0.586 (0.266–1.291)	1.759	0.185	–	–	–
	> 50	28	16						
Gender	Male	33	25	0.501 (0.068–3.695)	0.460	0.497	–	–	–
	Female	3	1						
Tumor size (cm)	≤ 5 cm	18	7	2.393 (1.003–5.710)	3.867	***0.049***	NS		
	> 5 cm	18	19						
Cirrhosis	No	25	17	1.203 (0.536–2.700)	0.201	0.654	–	–	–
	Yes	11	9						
Portal vein Tumor thrombus	No	34	17	4.275 (1.852–9.869)	11.585	***0.001***	2.905 (1.159–7.280)	5.179	***0.023***
	Yes	2	9						
Child-Pugh class	A	32	20	1.842 (0.737–4.606)	1.708	0.191	–	–	–
	B	4	6						
HBsAg	Negative	6	5	0.944 (0.356–2.504)	0.014	0.907	–	–	–
	Positive	30	21						
AFP (μg/L)	≤ 400	28	15	2.181 (0.996–4.776)	3.798	***0.051***	NS		
	> 400	8	11						
CEA (μg/L)	≤ 5	32	22	1.311 (0.451–3.808)	0.247	0.619	–	–	–
	> 5	4	4						
CA199 (kU/L)	≤ 34	27	16	1.699 (0.770–3.751)	1.720	0.190	–	–	–
	> 34	9	10						
Total CTCs	Negative	21	6	2.950 (1.184–7.349)	5.393	***0.020***	NS		
	Positive	15	20						
Mixed CTCs	Negative	25	9	2.935 (1.306–6.594)	6.797	***0.009***	NS		
	Positive	11	17						
Mesenchymal CTCs	Negative	29	8	4.740 (2.041–11.010)	13.099	***0.000***	3.453 (1.393–8.559)	7.157	***0.007***
	Positive	7	18						
Edmondson stage	I–II III–IV	35 1	23 3	0.343 (0.103–1.148)	3.011	***0.083***	NS		
BCLC stage	0 + A	14	9	1.362 (0.606–3.060)	0.560	0.454	–	–	–
	B + C	22	17						
ALP (U/L)	≤ 160	32	19	2.178 (0.906–5.236)	3.025	***0.082***	NS		
	> 160	4	7						
ALT (U/L)	≤ 75	27	22	0.656 (0.225–1.907)	0.601	0.438	–	–	–
	> 75	9	4						

*P < 0.10 will be further multivariate analysis

*NS* non-significance, *AFP* alpha-fetoprotein, *HBsAg* hepatitis B surface antigen, *CEA* carcinoembryonic antigen, *CA199* carbohydrate antigen 199, *BCLC* stage Barcelona Clinic Liver Cancer stage, *ALP* alkaline phosphatase, *ALT*, alanine aminotransferase

## Discussion

Currently, a large number of studies have demonstrated that epithelial-mesenchymal transition (EMT) plays a key role in tumor recurrence and metastasis.^13^,^14^ According to the EMT process, CTCs can be divided into different subtypes, including epithelial CTCs, mesenchymal CTCs, and mixed (epithelial/mesenchymal) CTCs.^15^ However, many techniques, including the CellSearch system, detect and isolate CTCs based only on epithelial markers, which is most likely to overlook the subpopulations of CTCs with undergoing EMT.^16^,^17^ An increasing number of studies have shown that tumor cells expressing mesenchymal markers lead to a poor prognosis for many tumors.^18^,^19^ To our knowledge, there is a lack of studies on the correlation between mesenchymal CTCs and postoperative HCC recurrence. Therefore, mesenchymal CTCs may be an ideal biomarker for predicting recurrence after radical resection of HCC. Moreover, the detection of CTCs can be used as a method for early intervention of HCC after radical resection. Thus, we used the second-generation CanPatrol™ CTC detection technology to isolate, identify, and classify CTCs in HCC patients. This technology can be used to classify CTCs in the peripheral blood into three categories based on EMT phenotype and to study the correlations between different CTC subtypes and ER of HCC.^20^

The present study examined the correlation between CTC phenotypes and early postoperative HCC recurrence, which was also the first study on the correlation between mesenchymal CTCs and HCC prognosis. It was demonstrated that total CTCs (*P* = 0.011), mesenchymal CTCs (*P* < 0.001), and mixed CTCs (*P* = 0.027) were positively correlated with postoperative recurrence through a rank-sum test. We found that the cutoff values for each CTC subtype to be positively correlated with recurrence through ROC curve analysis, with a definition of positive values for each CTC subtype (CTCs ≥ 4, mesenchymal CTCs ≥ 1, mixed CTCs ≥ 3). Cox regression analysis showed that the risk of ER was significantly higher in mesenchymal CTC-positive patients than in mesenchymal CTC-negative patients (HR = 3.453, *P* = 0.007). Meanwhile, a K-M test showed significantly shortened postoperative disease-free survival in mesenchymal CTC-positive patients (*P* < 0.001).

The mechanisms underlying the formation of recurrent lesions by CTCs have been shown to be closely related to EMT and mesenchymal-epithelial transition (MET).^18^,^21^ Tumor cells generate highly invasive mesenchymal CTCs in the peripheral blood through the EMT process.^22^ Although the majority of mesenchymal CTCs are cleared by the immune system, a small number of mesenchymal CTCs escape from immune surveillance and remain in a dormant state. These mesenchymal CTCs undergo the MET process following changes in the body’s immune activity and upon encountering specific microenvironments, resulting in recurrent colonization foci.^23^,^24^ According to the CTC formation hypothesis, tumor cells are spontaneously released into the peripheral blood during diagnostic or therapeutic procedures, leading to distant metastasis or intrahepatic recurrence when they return to the residual liver tissue.^25^ Before surgery, the patients were in a relatively stable state, which also meant their immune system was intact. Under such a condition, the internal environment was not considered suitable for CTC migration and colonization. However, due to the squeezing stimulation to the primary tumor foci during the operation, the tumor-neighboring microenvironment was damaged, which could lead to the shedding of a large number of tumor cells into the blood.^26^ In addition, postoperative internal environment imbalance could induce weakened ability of CTC clearance, which facilitates the colonization of highly invasive mesenchymal CTCs followed by tumor recurrence and metastasis.^24^ That is to say, patients who are preoperatively negative for CTCs may become postoperatively positive for CTCs due to surgical stimulation. Furthermore, the body is in a traditional “tumor-free” state after radical tumor resection. Accordingly, postoperative assessment of the relationship between CTCs and the recurrence of HCC not only allows relatively accurate predictions regarding recurrence but also excludes the impact of solid tumors per se on recurrence. Therefore, it is suggested that postoperative examination of the peripheral blood mesenchymal CTCs is more meaningful. Moreover, in prior studies of the relationship between CTCs in postoperative peripheral blood and tumor recurrence, CTCs were commonly collected within 4–5 weeks after surgery.^27^,^28^ Accordingly, we collected peripheral blood from HCC patients within 5 weeks after surgery to analyze the relationships between various CTC phenotypes and early recurrence. Our results further confirmed that postoperative mesenchymal CTCs were positively correlated with HCC recurrence and that the disease-free survival was significantly shortened in mesenchymal CTC-positive patients compared to mesenchymal CTC-negative patients.

Present results suggest that mesenchymal CTCs are not only associated with HCC recurrence but is also helpful in the prediction of early recurrence. Therefore, examination of peripheral mesenchymal CTCs in postoperative HCC patients can be a good secondary indicator in monitoring the prognosis of HCC patients, especially when traditional monitoring methods cannot provide evidence for the diagnosis. This method could improve the early diagnosis of postoperative HCC recurrence, facilitate early personalized interventions in postoperative patients with positive results for mesenchymal CTCs, and prolong their survival.^21^,^29^ In addition, CTCs of different subtypes have different properties and show different responses to the same treatment.^30^ So, for HCC patients who are postoperatively positive for mesenchymal CTCs, we can eliminate mesenchymal CTCs in the peripheral blood via chemotherapy and other treatments to effectively block the formation of recurrence after surgery and thereby achieve the early prevention of HCC recurrence.^31^This finding may provide new ideas for the development of new medicines that act on mesenchymal CTCs. Therefore, in future studies, we will also focus on drugs that can effectively reduce CTCs in the peripheral blood of patients with HCC to prevent early recurrence.

As described above, CTCs detected after radical resection of a tumor might be derived from two sources: (1) CTCs that were preoperatively present in peripheral blood and (2) CTCs produced when tumor cells were torn from tumor foci and released into the circulating blood due to mechanical stimulation of these foci and changes in the environment of the body during surgery. And the formation of recurrent lesions is related to both the body’s immune status and the invasive ability of CTCs per se. In conjunction with our study results, it indicates that mesenchymal CTCs may largely arise from a CTC subtype with strong invasion and colonization capabilities. Therefore, we will further study the functional characteristics and properties of mesenchymal CTCs in HCC to elucidate the underlying role of mesenchymal CTCs in the molecular mechanism of recurrent foci formation in HCC. On the basis of this study, we will conduct a prospective, multicenter clinical study to further validate the relationship between mesenchymal CTCs and HCC prognosis and further analyze the effects of surgical procedure on CTC phenotypes and quantities to optimize the surgical procedure.

## Conclusions

In conclusion, we found that postoperative total CTCs, mixed CTCs, and mesenchymal CTCs count was significantly higher in recurrence patients of hepatocellular carcinoma than in non-recurrence patients, and there is no difference in postoperative epithelial CTC counts between the two groups. Furthermore, we demonstrated that postoperative mesenchymal CTC-positive in the peripheral blood indicates early recurrence of hepatocellular carcinoma after curative resection. Monitoring postoperative mesenchymal CTCs may be a promising predictor of early recurrence of HCC, and eradicating these cells might open a therapeutic avenue toward preventing HCC recurrence.
